# Combined use of serum (1,3)-β-d-glucan and procalcitonin for the early differential diagnosis between candidaemia and bacteraemia in intensive care units

**DOI:** 10.1186/s13054-017-1763-5

**Published:** 2017-07-10

**Authors:** Daniele Roberto Giacobbe, Malgorzata Mikulska, Mario Tumbarello, Elisa Furfaro, Marzia Spadaro, Angela Raffaella Losito, Alessio Mesini, Gennaro De Pascale, Anna Marchese, Marco Bruzzone, Paolo Pelosi, Michele Mussap, Alexandre Molin, Massimo Antonelli, Brunella Posteraro, Maurizio Sanguinetti, Claudio Viscoli, Valerio Del Bono, on behalf of ISGRI-SITA (Italian Study Group on Resistant Infections of the Società Italiana Terapia Antinfettiva)

**Affiliations:** 1 Infectious Diseases Division, University of Genoa (DISSAL) and Ospedale Policlinico San Martino - IRCCS per l’Oncologia, L.go R. Benzi, 10 - 16132, Genoa, Italy; 2 Institute of Infectious Diseases, Università Cattolica del Sacro Cuore, Fondazione Policlinico Universitario Agostino Gemelli, Rome, Italy; 3 Department of Intensive Care and Anesthesiology, Università Cattolica del Sacro Cuore, Fondazione Policlinico Universitario Agostino Gemelli, Rome, Italy; 4 Microbiology Unit, University of Genoa (DISC) and Ospedale Policlinico San Martino - IRCCS per l’Oncologia, Genoa, Italy; 5 Clinical Epidemiology Unit, Ospedale Policlinico San Martino - IRCCS per l’Oncologia, Genoa, Italy; 6 Anesthesiology and Intensive Care Unit, DIPEA, Ospedale Policlinico San Martino - IRCCS per l’Oncologia, Genoa, Italy; 7 Anesthesiology and Intensive Care, University of Genoa (DISC), Genoa, Italy; 8 Department of Bio-medical Laboratory, Ospedale Policlinico San Martino - IRCCS per l’Oncologia, Genoa, Italy; 9 Institute of Public Health (Section of Hygiene), Università Cattolica del Sacro Cuore, Fondazione Policlinico Universitario Agostino Gemelli, Rome, Italy; 10 Institute of Microbiology, Università Cattolica del Sacro Cuore, Fondazione Policlinico Universitario Agostino Gemelli, Rome, Italy

**Keywords:** *Candida*, Bloodstream infections, BSI, Sepsis, Fungal antigens, Non-culture-based methods, Biomarker, Critically ill patients

## Abstract

**Background:**

This study aimed to assess the combined performance of serum (1,3)-β-d-glucan (BDG) and procalcitonin (PCT) for the differential diagnosis between candidaemia and bacteraemia in three intensive care units (ICUs) in two large teaching hospitals in Italy.

**Methods:**

From June 2014 to December 2015, all adult patients admitted to the ICU who had a culture-proven candidaemia or bacteraemia, as well as BDG and PCT measured closely to the time of the index culture, were included in the study. The diagnostic performance of BDG and PCT, used either separately or in combination, was assessed by calculating the sensitivity, specificity, positive predictive value (PPV), negative predictive value (NPV), and positive and negative likelihood ratios (LR+ and LR–). Changes from pre-test probabilities to post-test probabilities of candidaemia and bacteraemia were inferred from Fagan’s nomograms.

**Results:**

One hundred and sixty-six patients were included, 73 with candidaemia (44%) and 93 with bacteraemia (56%). When both markers indicated candidaemia (BDG ≥80 pg/ml and PCT <2 ng/ml) they showed higher PPV (96%) compared to 79% and 66% for BDG or PCT alone, respectively. When both markers indicated bacteraemia (BDG <80 pg/ml and PCT ≥2 ng/ml), their NPV for candidaemia was similar to that of BDG used alone (95% vs. 93%). Discordant BDG and PCT results (i.e. one indicating candidaemia and the other bacteraemia) only slightly altered the pre-test probabilities of the two diseases.

**Conclusions:**

The combined use of PCT and BDG could be helpful in the diagnostic workflow for critically ill patients with suspected candidaemia.

**Electronic supplementary material:**

The online version of this article (doi:10.1186/s13054-017-1763-5) contains supplementary material, which is available to authorized users.

## Background

Differentiating candidaemia from bacteraemia is a tough diagnostic challenge for clinicians worldwide, mainly because of their very similar clinical presentation [1–4]. Although blood cultures remain the mainstay for a definite aetiological diagnosis, their low sensitivity and slow turn-around time have prompted the development of rapid antigen-based diagnostic methods, such as the (1,3)-β-d-glucan (BDG) assay, as useful tools for anticipating the diagnosis of candidaemia and especially for excluding it, strengthening the diagnosis of bacteraemia and favouring the safe discontinuation of useless antifungal treatments [5–8].

In addition to serum fungal antigens, other less specific markers might help clinicians in the differential diagnosis between candidaemia and bacteraemia. For example, serum procalcitonin (PCT), a well-recognized marker of bacteraemia, has been suggested to be normal or only slightly increased in candidaemia [9–13]. However, a still unanswered question is how to interpret and balance the results of PCT and BDG when used in combination for the differential diagnosis between the two diseases.

The primary objective of this study was to assess the combined performance of PCT and BDG for the differential diagnosis between candidaemia and bacteraemia in critically ill patients in the intensive care unit (ICU).

## Methods

This multicentre retrospective study was conducted in three adult ICU wards of two large Italian tertiary care centres; two in Ospedale Policlinico San Martino, a 1200-bed teaching hospital in Genoa, and one in Policlinico Agostino Gemelli, a 1400-bed teaching hospital in Rome. In both hospitals, BDG testing is routinely used for diagnostic purposes in patients with signs and symptoms of infection and considered at risk of candidaemia according to clinical judgement.

From June 2014 to December 2015, all critically ill patients with candidaemia or bacteraemia fulfilling the following criteria were included in the study: 1) 18 years of age or older; 2) BDG testing performed within 48 h before or after the time of the index culture; and 3) serum PCT measured within 24 h before or after the time of the index culture. The different window of time for BDG and PCT was arbitrarily selected in view of the different kinetics in the blood of the two markers with respect to the infectious syndrome [14, 15]. The time of the index culture was defined as the day on which the first blood culture, which later resulted positive for *Candida* spp. or bacteria, was drawn. Patients with mixed infections (defined as presence of positive cultures for *Candida* spp. and bacteria less than 48 h apart) were excluded from the study.

### Definitions of candidaemia and bacteraemia

Candidaemia and bacteraemia were defined as the presence of at least one blood culture positive for *Candida* or bacteria, respectively, in presence of signs and symptoms of infection [16, 17]. For coagulase-negative staphylococci and other common skin contaminants, at least two consecutive blood cultures positive for the same pathogen were deemed necessary to define bacteraemia [18].

### Data collected for the analysis

The following demographic and clinical characteristics were retrieved from medical records and laboratory databases at the time of candidaemia or bacteraemia: age, gender, type of patient (surgical vs. medical), previous abdominal surgery (within 30 days), chronic renal failure, diabetes mellitus, Charlson comorbidity score [19], presence of a central venous catheter (CVC) from more than 48 h, haemodialysis, receipt of corticosteroids, receipt of albumin, receipt of immunoglobulins, previous antibiotic therapy (within 30 days), previous antifungal therapy (within 30 days), *Candida* colonization (defined as one or more *Candida*-positive cultures from non-sterile sites), and length of hospital stay prior to the onset of candidaemia or bacteraemia.

### Laboratory

BDG evaluation was performed with the Fungitell assay (Associates of Cape Cod, Cape Cod, MA, USA). BDG samples were stored at –20 °C, and assays were performed according to the manufacturer’s recommendations. Values lower than 80 pg/ml and higher than 500 pg/ml were reported as <80 pg/ml and >500 pg/ml, respectively.

Procalcitonin (PCT) was measured by a quantitative sandwich chemiluminescence immunoassay (CLIA; Liaison® BRAHMS® PCT II GEN, DiaSorin, Saluggia, Italy) optimized on an automated analytical platform (Liaison® XL, DiaSorin, Saluggia, Italy) [20]. The method is based on a specific monoclonal antibody coated on the magnetic particles; a second antibody is linked to an isoluminol-conjugate antibody. After the first incubation, the solid phase is added to the reaction and in the presence of PCT a sandwich is formed. The intra- and inter-assay imprecision, expressed as coefficient of variation (CV, %) ranged 1.0–4.8% and 3.5–16.4%, respectively, depending on the PCT concentration.

Identification of bacteria and *Candida* spp. was performed by the VITEK 2 automated system (bioMérieux, Marcy l’Etoile, France) or by MALDI-TOF mass spectrometry (Bruker Daltonik, Bremen, Germany), in accordance with the laboratory diagnostic procedure adopted in Genoa and in Rome, respectively.

### Statistical analysis

Categorical variables are presented as absolute frequencies and percentages, while continuous variables are presented as median value and interquartile range (IQR).

The collected demographic and clinical characteristics of patients were tested for their association with candidaemia or bacteraemia by univariable analysis, by means of the chi-square test, the Fisher exact test, or the Mann-Whitney *U* test, as appropriate.

The diagnostic performance of BDG and PCT used separately for differentiating between the two diseases was assessed by calculating their sensitivity, specificity, positive predictive value (PPV), negative predictive value (NPV), positive likelihood ratio (LR+), and negative likelihood ratio (LR–) for the diagnosis of candidaemia over bacteraemia in our population, at different cut-off levels. Among the explored thresholds, we also included the standard BDG and PCT thresholds (80 pg/ml and 0.5 ng/ml) reported in the literature for the diagnosis of candidaemia per se and bacteraemia per se, respectively [9, 21]. The optimal BDG and PCT cut-offs in our population were defined as the points with the maximum Youden Index on the respective receiver-operating characteristic (ROC) curves.

To verify that both BDG and PCT were independently associated with the diagnosis of candidaemia over bacteraemia, their serum values were dichotomised according to their optimal cut-offs, and included in a multivariable stepwise backward logistic regression along with demographic and clinical factors showing an association with candidaemia in univariable comparisons (*p* < 0.10). Subsequently, their combined performance for the diagnosis of candidaemia over bacteraemia was reported in terms of sensitivity, specificity, PPV, NPV, LR+, and LR–.

Finally, changes from the pre-test probability (approximating to the prevalence of disease by assuming a similar individual risk among tested patients) to the post-test probability of candidaemia and bacteraemia (the latter defined as 1 – probability of candidaemia) were inferred from Fagan’s nomograms [22] for either BDG and PCT used alone or in combination.

The analyses were performed using SPSS Statistics version 21.0 (IBM Corp., Armonk, NY, USA) and R Statistical Software version 3.3.0 (R Foundation for Statistical Computing, Vienna, Austria).

## Results

**Fig. 1:**
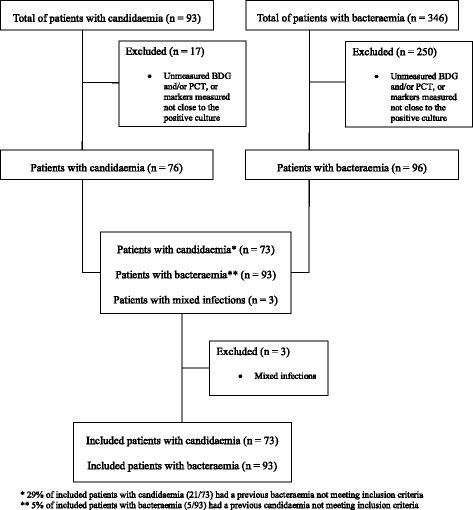
Flow chart of the patient inclusion process. *BDG* (1,3)-β-d-glucan, *PCT* procalcitonin

During the study period, 166 patients were included in the study (Fig. 1). Of these, 73 had candidaemia and 93 bacteraemia (44% and 56%, respectively). The complete demographic and clinical characteristics of the patients, as well as their possible association with candidaemia or bacteraemia in univariable analysis, are reported in Table 1. Patients with candidaemia were more likely than those with bacteraemia to have a CVC (82% vs. 65%), have been treated with antibiotics (88% vs. 49%), and have a longer hospital stay before the development of the defining infection (median 21 vs. 14 days). The other baseline characteristics were not significantly different between the two populations.

**Table 1:** Demographic and clinical characteristics of patients with candidaemia and bacteraemia at the time of infection

Variable	Candidaemia* (*n* = 73, 44%)	Bacteraemia** (*n* = 93, 56%)	*P*
Demographic variables			
Age, years (median (IQR))	64 (52–76)	67 (48–73)	0.439
Male gender	43 (59)	54 (58)	0.913
Clinical variables			
Type of patient			0.502
Surgical	28 (38)	31 (33)	
Medical	45 (62)	62 (67)	
Previous abdominal surgery	13 (18)	16 (17)	0.919
Chronic renal failure	19 (26)	23 (25)	0.849
Diabetes mellitus	14 (19)	20 (22)	0.712
Charlson score (median (IQR))	3 (2-4)	2 (2-5)	0.546
Presence of CVC	60 (82)	60 (65)	0.012
Haemodialysis	7 (10)	13 (14)	0.389
Receipt of albumin	6 (8)	17 (18)	0.073
Receipt of immunoglobulins	0 (0)	2 (2)	0.504
Corticosteroid therapy	15 (21)	17 (18)	0.713
Previous antibiotics	64 (88)	46 (49)	<0.001
Previous antifungals	12 (16)	8 (9)	0.124
*Candida* colonization	21 (29)	23 (25)	0.599
Time from admission to candidaemia or bacteraemia, days (median (IQR))	21 (10–46)	14 (8–29)	<0.001

Values are shown as *n* (%) unless otherwise indicated

* *Candida albicans* (*n* = 37), *Candida parapsilosis* (*n* = 23), *Candida tropicalis* (*n* = 7), *Candida glabrata* (*n* = 4), *Candida guilliermondii* (*n* = 1), *Candida lusitaniae* (*n* = 1)

** *Klebsiella* spp. (*n* = 23), *Staphylococcus aureus* (*n* = 12), *Escherichia coli* (*n* = 11), *Pseudomonas aeruginosa* (*n* = 11), coagulase-negative staphylococci (*n* = 8), *Enterococcus* spp. (*n* =5), *Enterobacter* spp. (*n* = 4), *Proteus* spp. (*n* = 4), *Serratia* spp. (*n* = 3), *Acinetobacter* spp. (*n* = 2), *Streptococcus* spp. (*n* = 2), *Citrobacter* spp. (*n* = 1), *Providencia* spp. (*n* = 1), *Pseudomonas aeruginosa* + *Staphylococcus aureus* (*n* = 1), *Acinetobacter* spp. + *Pseudomonas aeruginosa* (*n* = 1), *Acinetobacter* spp. + *Staphylococcus aureus* (*n* = 1), *Enterococcus* spp. + *Klebsiella* spp. (*n* = 1), *Escherichia coli* + *Klebsiella* spp. (*n* = 1), *Escherichia coli* + *Pseudomonas aeruginosa* (*n* = 1)

*CVC* central venous catheter, *IQR* interquartile range

**Fig. 2:**
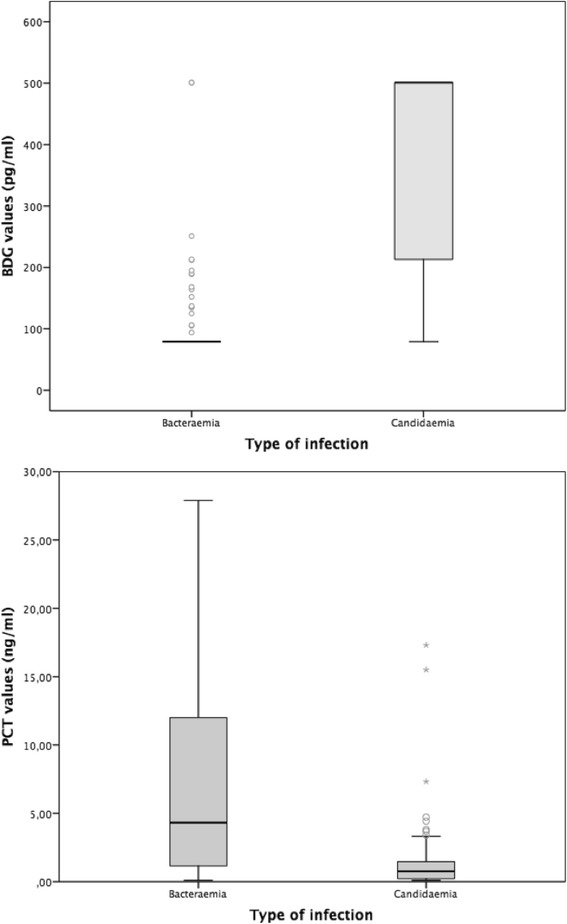
Distribution of BDG and PCT serum levels in patients with bacteraemia or candidaemia. Box plots of the distribution of serum (1,3)-β-d-glucan (*BDG*) and procalcitonin (*PCT*) in study patients, divided according to the type of infection. BDG values lower than 80 pg/ml and higher than 500 pg/ml were considered as 79 pg/ml and 501 pg/ml, respectively. Outliers for PCT values >30 ng/ml are not displayed in the graph

**Fig. 3:**
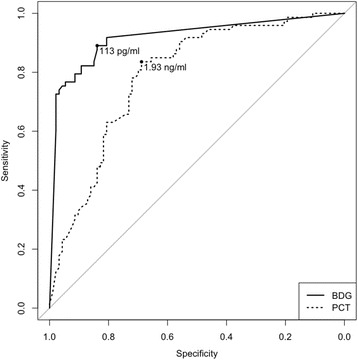
Performance of BDG and PCT for the diagnosis of candidaemia over bacteraemia in the study population. The area under the ROC curve was 0.919 for (1,3)-β-d-glucan (*BDG*) (95% CI 0.875–0.962) and 0.789 for procalcitonin (*PCT*) (95% CI 0.720–0.856). The points on the curves with the maximum Youden Index were 113 pg/ml for BDG and 1.93 ng/ml for PCT. Diagnostic performances of the two markers used alone or combined are detailed in Tables 2 and 3, respectively

The distribution of BDG and PCT values in patients with bacteraemia and candidaemia is shown in Fig. 2. Most patients were tested for BDG (139/166, 84%) and PCT (143/166, 86%) within 12 h before and 24 h after the positive blood draw. Patients with candidaemia had higher BDG values (median >500 vs. <80 pg/ml, *p* < 0.001) and lower PCT values (median 0.76 vs. 4.32 ng/ml, *p* < 0.001) than those with bacteraemia. The optimal discriminatory cut-offs of BDG and PCT for diagnosing candidaemia over bacteraemia in our population were BDG ≥113 pg/ml and PCT <1.93 ng/ml, respectively (Fig. 3). As shown in Table 2, the performance of the optimal BDG cut-off of ≥113 pg/ml (89% sensitivity, 84% specificity, 81% PPV, 91% NPV) was very similar to that of the standard BDG cut-off of ≥80 pg/ml (92% sensitivity, 81% specificity, 79% PPV, 93% NPV), whereas the optimal PCT cut-off of <1.93 ng/ml (84% sensitivity, 69% specificity, 68% PPV, 84% NPV) performed better in terms of sensitivity and specificity for differentiating between the two diseases than a PCT cut-off of <0.5 ng/ml (41% sensitivity, 86% specificity, 70% PPV, 65% NPV). Although with the limits of subgroup analyses and without detecting a statistically significant difference, it is of note that BDG specificity was apparently lower in patients with *Candida* colonization than in those without (78% vs. 86%, *p* = 0.514), whereas its sensitivity was similar in those who received antifungals previously and in those who did not (92%% vs 89%%, *p* = 1). In the multivariable analysis of factors associated with the diagnosis of candidaemia over bacteraemia, BDG ≥113 pg/ml (odds ratio (OR) 31.0, 95% confidence interval (CI) 11.0–87.7; *p* < 0.001) and PCT <1.93 ng/ml (OR 9.5, 95% CI 3.3–27.1; *p* < 0.001) were the only two variables independently associated with the diagnosis.

**Table 2:** Sensitivity, specificity, PPV, NPV, LR+, and LR– of BDG and PCT used separately for the diagnosis of candidaemia over bacteraemia in the study population

	No./total patients							
Cut-off	Candidaemia (TP/total)	Bacteraemia (TN/total)	Sensitivity (%)	Specificity (%)	PPV (%)	NPV (%)	LR+	LR–
BDG								
≥80 pg/ml	67/73	75/93	92	81	79	93	4.74	0.10
≥100 pg/ml	65/73	76/93	89	82	79	90	4.87	0.13
≥113 pg/ml	65/73	78/93	89	84	81	91	5.52	0.13
≥120 pg/ml	65/73	78/93	89	84	81	91	5.52	0.13
≥140 pg/ml	60/73	82/93	82	88	85	86	6.95	0.20
≥160 pg/ml	60/73	83/93	82	89	86	86	7.64	0.20
≥180 pg/ml	57/73	85/93	78	91	88	84	9.08	0.24
≥200 pg/ml	55/73	88/93	75	95	92	83	14.01	0.26
PCT								
<0.50 ng/ml	30/73	80/93	41	86	70	65	2.94	0.68
<1.00 ng/ml	46/73	75/93	63	81	72	74	3.26	0.46
<1.93 ng/ml	61/73	64/93	84	69	68	84	2.68	0.24
<2.00 ng/ml	61/73	62/93	84	67	66	84	2.51	0.25
<3.00 ng/ml	63/73	54/93	86	58	62	84	2.06	0.24
<4.00 ng/ml	67/73	49/93	92	53	60	89	1.94	0.16
<5.00 ng/ml	69/73	43/93	95	46	58	91	1.76	0.12

*BDG* (1,3)-β-d-glucan, *LR+* positive likelihood ratio, *LR–* negative likelihood ratio, *NPV* negative predictive value, *PCT* procalcitonin, *PPV* positive predictive value, *TN* true negatives, *TP* true positives

**Fig. 4:**
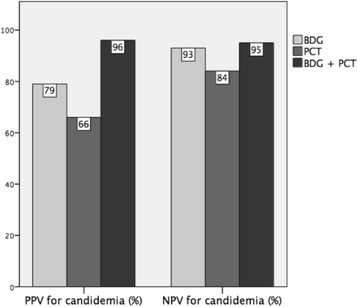
Positive predictive value (*PPV*) and negative predictive value (*NPV*) for candidaemia of (1,3)-β-d-glucan (*BDG*) and procalcitonin (*PCT*) considered both separately and in combination. Cut-offs for candidaemia used for the comparisons in the graph are ≥80 pg/ml for BDG and <2 ng/ml for PCT. For the combination (BDG + PCT), the reported PPV for candidaemia (also readable as NPV for bacteraemia) was obtained when both markers were concordant in indicating candidaemia (BDG ≥80 pg/ml and PCT <2 ng/ml), while the reported NPV for candidaemia (also readable as PPV for bacteraemia) was obtained when both markers when concordant in indicating bacteraemia (BDG <80 pg/ml and PCT ≥2 ng/ml)

We then assessed the diagnostic performance of BDG and PCT used in combination for the diagnosis of candidaemia over bacteraemia in our cohort. For practical purposes, we tested the combination of the widely used standard BDG cut-off of ≥80 pg/ml (since its performance was very similar to that of the optimal cut-off of ≥113 pg/ml) and the optimal PCT cut-off of <1.93 ng/ml rounded to the nearest ng/ml unit (i.e. <2 ng/ml). Sensitivity, specificity, PPV, NPV, LR+, and LR– of BDG and PCT used in combination for diagnosing candidaemia over bacteraemia are summarized in Table 3. As reported in Table 3, and graphically in Fig. 4, when both markers were indicative of candidaemia (BDG ≥80 pg/ml and PCT <2 ng/ml) they showed a higher PPV for candidaemia (96%) compared to 79% and 66% when BDG and PCT were used separately, respectively. In contrast, when both markers suggested the absence of candidaemia (i.e. when they were both indicative of bacteraemia, BDG <80 pg/ml and PCT ≥2 ng/ml), their NPV for candidaemia was very similar to that of BDG used alone (95% vs. 93%, respectively).

**Table 3:** Sensitivity, specificity, PPV, NPV, LR+, and LR– of BDG and PCT used in combination for the diagnosis of candidaemia over bacteraemia in the study population

	No./total patients							
Cut-off	Candidaemia (TP/total)	Bacteraemia (TN/total)	Sensitivity (%)	Specificity (%)	PPV (%)	NPV (%)	LR+	LR–
BDG ≥80 pg/ml and/or PCT <2 ng/ml	70/73	56/93	96	60	65	95	2.41	0.07
BDG ≥80 pg/ml and PCT <2 ng/ml	48/73	91/93	66	98	96	78	30.58	0.35

*BDG* (1,3)-β-d-glucan, *LR+* positive likelihood ratio, *LR–* negative likelihood ratio, *NPV* negative predictive value, *PCT* procalcitonin, *PPV* positive predictive value, *TN* true negatives, *TP* true positives

To further characterize the diagnostic performance of BDG and PCT in combination, including the effect of discordant results (i.e. when one marker indicated candidaemia and the other bacteraemia), we used Fagan’s nomograms (Additional file 1: Figure S1). Specifically, Additional file 1 (Figure S1) shows the pre-test and post-test probabilities of candidaemia according to different BDG and PCT results (considered both separately and in combination), at the prevalence of the disease registered in our study (44%). The pre-test and post-test probability of bacteraemia can be calculated as 1 minus the pre-test or post-test probability of candidaemia, respectively. Post-test probabilities of candidaemia and bacteraemia at prevalences different from that registered in our study can be inferred manually on Fagan’s nomograms by intersecting the likelihood ratio (LR) of candidaemia with a straight line starting from the selected pre-test probability of the disease (some examples are provided in Additional file 2, which also propose a possible therapeutic algorithm to be validated in confirmatory studies).

## Discussion

In this retrospective study in a cohort of critically ill patients with candidaemia or bacteraemia, the combined use of BDG and PCT performed better than the two markers used separately for identifying true cases of candidaemia, in the case of concordant results indicating the disease. Conversely, when both markers indicated bacteraemia, their performance was similar to that of BDG used alone, highlighting the high NPV for candidaemia of the BDG test.

Recently, Martínez-Jiménez and colleagues [23] reported on the performance of BDG for the diagnosis of candidaemia in a cohort of 81 patients with either candidaemia or bacteraemia, showing that the performance of the standard BDG cut-off of ≥80 pg/ml for diagnosing candidaemia over bacteraemia (84% sensitivity, 92% specificity, 87% PPV, 90% NPV) was consistent with that reported in the literature for the diagnosis of candidaemia per se [5–8, 24–29]. Our results are in line with these findings, since the overall performance of the optimal BDG cut-off for the diagnosis of candidaemia in our population (≥113 pg/ml) was very similar to that of the standard cut-off. In contrast, while a recent meta-analysis showed a PCT threshold of 0.5 ng/ml to be optimal for the diagnosis of bacteraemia [9], the same threshold was inadequate for differentiating bacteraemia from candidaemia in our study, and a higher value (1.93 ng/ml) performed far better for this purpose. This result is not surprising since patients with candidaemia might have slightly increased PCT values [11–13]. In this regard, the median PCT concentration of 0.73 ng/ml we found in patients with candidaemia is in line with values suggested by other authors for possibly indicating candidaemia [11–13]. However, it should be noted that in our population the discriminatory performance of PCT was overall inferior to that of BDG (sensitivity 84% and specificity 67% for PCT <2 ng/ml vs. sensitivity 92% and specificity 81% for BDG ≥80 pg/ml). For this reason, in our opinion, PCT should be used cautiously as a single biomarker for diagnosing or ruling out candidaemia when and where BDG is not available.

Our following step was to assess the performance of BDG and PCT used in combination for differentiating between the two diseases. In this regard, we think three possibly useful observations stemming from our study are: 1) in comparison with a positive BDG value considered alone, the presence of both BDG and PCT indicative of candidaemia might strengthen the clinician’s confidence in having identified a true episode of the disease, by increasing specificity and PPV from 81% and 79% to 98% and 96%, respectively; 2) the presence of both BDG and PCT indicative of bacteraemia does not particularly add to the already good ability of BDG alone in ruling out candidaemia, since sensitivity and NPV increased only from 92% and 93% to 96% and 95%, respectively; and 3) in case of discordant BDG and PCT values, the probabilities of candidaemia and bacteraemia might be not particularly altered by the results of the two tests. Given that, in our opinion, the choice on whether discontinuing antifungals in presence of a negative BDG plus low PCT values should be taken very cautiously, at least pending cultures results. Indeed, from a speculative standpoint, we might hypothesise that low PCT values could help unmask some of the rare false-negative BDG results, thus preventing from unsafely discontinuing antifungals. For example, negative BDG results could be found in some cases of *C. parapsilosis* candidaemia, as we observed in a previous single-centre experience also including non-ICU patients and a higher number of *C. parapsilosis* isolates [6], albeit this data has to be confirmed in further, prospective studies. However, it remains clear that no definite therapeutic conclusions can be drawn from the present preliminary two-centre retrospective experience, and validation is warranted.

The main drawback of our study is its retrospective nature. Among limitations of the retrospective design is that blood samples for BDG and PCT testing were not collected at standardised points in time. Consequently, some measured values might be different from those present at the time of the index culture, although this bias is reduced by the fact that we mainly included patients who underwent BDG and PCT testing very close to the positive blood draw. Our aim was indeed to include only patients at risk of candidaemia who underwent BDG testing in the presence of signs and symptoms of the disease and pending cultures results, therefore reflecting the diagnostic use of the marker in real life. However, it should also be considered that we could not always retrospectively explore why clinicians suspected the disease. Consequently, we cannot be sure that the high pre-test probability of candidaemia observed in our cohort (44%) reflected only an appropriate use of BDG or was also dependent on a selection bias, and thus unrepresentative of the true prevalence of candidaemia in patients at risk. Furthermore, although it is overall reasonable to approximate the pre-test probability of a certain disease to its local prevalence in selected populations, the former is inherently individual and might increase or decrease because of individual conditions and factors [30]. Sensitivity and specificity of a marker (and thus the LR of a certain disease) might also not always be fixed irrespective of prevalence, a fact that should be considered when extrapolating our results to other prevalences of candidaemia on Fagan’s nomograms [31]. Another limitation is that we did not include mixed infections (candidaemia plus bacteraemia). Although their occurrence was uncommon in our cohort, and thus unlikely to influence the overall diagnostic performance of the combination, this might not be the rule in other hospitals and settings [32]. The low rate of mixed infections we detected might also reflect the short window of time we used for defining an infection as concomitant for diagnostic purposes. Finally, an important aspect should be taken into account when interpreting our results, that is they apply only to critically ill patients at risk of candidaemia, and cannot be extrapolated to critically ill patients without risk factors for fungal infections. However, in our opinion restricting the analysis to patients at risk should be ultimately viewed more as a strength than a limitation.

## Conclusions

The combined use of PCT and BDG could be helpful in the diagnostic workflow for critically ill patients with suspected candidaemia. Further studies are needed to understand whether this might significantly impact current therapeutic algorithms, as well as be a cost-effective strategy.

## Additional files

Additional file 1: Fagan’s nomograms of pre-test and post-test probability of candidaemia according to BDG and PCT results. (PDF 408 kb)

Additional file 2: Example of a possible therapeutic model based on Fagan’s nomograms of pre-test and post-test probability of candidaemia and bacteraemia according to BDG and PCT results. (PDF 382 kb)

## Abbreviations

BDG: (1,3)-β-d-glucan
CI: Confidence interval
CVC: Central venous catheter
ICU: Intensive care unit
IQR: Interquartile range
LR–: Negative likelihood ratio
LR+: Positive likelihood ratio
NPV: Negative predictive value
OR: Odds ratio
PCT: Procalcitonin
PPV: Positive predictive value
ROC: Receiver-operating characteristic

## References

[1] Fernandez J, Erstad BL, Petty W, Nix DE (2009). Time to positive culture and identification for Candida bloodstream infections. Diagn Microbiol Infect Dis.

[2] Morrell M, Fraser VJ, Kollef MH (2005). Delaying the empiric treatment of candida bloodstream infection until positive blood culture results are obtained: a potential risk factor for hospital mortality. Antimicrob Agents Chemother.

[3] Clancy CJ, Nguyen MH (2013). Finding the “missing 50%” of invasive candidiasis: how nonculture diagnostics will improve understanding of disease spectrum and transform patient care. Clin Infect Dis.

[4] Giacobbe DR, Esteves P, Bruzzi P, Mikulska M, Furfaro E, Mesini A (2015). Initial serum (1,3)-beta-D-glucan as a predictor of mortality in proven candidaemia: findings from a retrospective study in two teaching hospitals in Italy and Brazil. Clin Microbiol Infect.

[5] Posteraro B, De Pascale G, Tumbarello M, Torelli R, Pennisi MA, Bello G (2011). Early diagnosis of candidaemia in intensive care unit patients with sepsis: a prospective comparison of (1/3)-β-d-glucan assay, Candida score, and colonization index. Crit Care.

[6] Mikulska M, Giacobbe DR, Furfaro E, Mesini A, Marchese A, Del Bono V (2016). Lower sensitivity of serum (1,3)-beta-D-glucan for the diagnosis of candidaemia due to Candida parapsilosis. Clin Microbiol Infect.

[7] Senn L, Robinson JO, Schmidt S, Knaup M, Asahi N, Satomura S (2008). 1,3-beta-d-Glucan antigenemia for early diagnosis of invasive fungal infections in neutropenic patients with acute leukemia. Clin Infect Dis.

[8] Del Bono V, Delfino E, Furfaro E, Mikulska M, Nicco E, Bruzzi P (2011). Clinical performance of the (1,3)-β-d-glucan assay in early diagnosis of nosocomial Candida bloodstream infections. Clin Vaccine Immunol.

[9] Hoeboer SH, van der Geest PJ, Nieboer D, Groeneveld AB (2015). The diagnostic accuracy of procalcitonin for bacteraemia: a systematic review and meta-analysis. Clin Microbiol Infect.

[10] Wacker C, Prkno A, Brunkhorst FM, Schiattmann P (2013). Procalcitonin as a diagnostic marker for sepsis: a systematic review and meta-analysis. Lancet Infect Dis.

[11] Cortegiani A, Russotto V, Montalto F, Foresta G, Accurso G, Palmeri C (2014). Procalcitonin as a marker of Candida species detection by blood culture and polymerase chain reaction in septic patients. BMC Anesthesiol.

[12] Martini A, Gottin L, Menestrina N, Schweiger V, Simion D, Vincent JL (2010). Procalcitonin levels in surgical patients at risk of candidaemia. J Infect.

[13] Charles PE, Dalle F, Aho S, Quenot JP, Doise JM, Aube H (2006). Serum procalcitonin measurement contribution to the early diagnosis of candidemia in critically ill patients. Intensive Care Med.

[14] Koo S, Baden LR, Marty FM (2012). Post-diagnostic kinetics of the (1,3)-β-D-glucan assay in invasive aspergillosis, invasive candidiasis and Pneumocystis jirovecii pneumonia. Clin Microbiol Infect.

[15] Charles PE, Tinel C, Barbar S, Aho S, Prin S, Doise JM (2009). Procalcitonin kinetics within the first days of sepsis: relationship with the appropriateness of antibiotic therapy and the outcome. Crit Care.

[16] De Pauw B, Walsh TJ, Donnelly JP, Stevens DA, Edwards JE, Calandra T (2008). Revised definitions of invasive fungal disease from the European Organization for Research and Treatment of Cancer/Invasive Fungal Infections Cooperative Group and the National Institute of Allergy and Infectious Diseases Mycoses Study Group (EORTC/MSG) Consensus Group. Clin Infect Dis.

[17] Russell JA (2006). Management of sepsis. N Engl J Med.

[18] Elzi L, Babouee B, Vögeli N, Laffer R, Dangel M, Frei R (2012). How to discriminate contamination from bloodstream infection due to coagulase-negative staphylococci: a prospective study with 654 patients. Clin Microbiol Infect.

[19] Charlson ME, Pompei P, Ales KL, MacKenzie CR (1987). A new method of classifying prognostic co-morbidity in longitudinal studies: development and validation. J Chronic Dis.

[20] Fortunato A (2016). A new sensitive automate assay for procalcitonin detection: Liaison® BRAHMS® PCT II GEN. Pract Lab Med.

[21] Cuenca-Estrella M, Verweij PE, Arendrup MC, Arikan-Akdagli S, Bille J, Donnelly JP (2016). ESCMID guideline for the diagnosis and management of Candida diseases 2012: diagnostic procedures. Clin Microbiol Infect.

[22] Fagan TJ (1975). Letter: nomogram for Bayes theorem. N Engl J Med.

[23] Martínez-Jiménez MC, Muñoz P, Valerio M, Alonso R, Martos C, Guinea J (2015). Candida biomarkers in patients with candidaemia and bacteraemia. J Antimicrob Chemother.

[24] Karageorgopoulos DE, Vouloumanou EK, Ntziora F, Michalopoulos A, Rafailidis PI, Falagas ME (2011). Beta-D-glucan assay for the diagnosis of invasive fungal infections: a meta-analysis. Clin Infect Dis.

[25] Lu Y, Chen YQ, Guo YL, Qin SM, Wu C, Wang K (2011). Diagnosis of invasive fungal disease using serum (1,3)-beta-D-glucan: a bivariate meta-analysis. Intern Med.

[26] Onishi A, Sugiyama D, Kogata Y, Saegusa J, Sugimoto T, Kawano S (2012). Diagnostic accuracy of serum 1,3-beta-D-glucan for pneumocystis jiroveci pneumonia, invasive candidiasis, and invasive aspergillosis: systematic review and meta-analysis. J Clin Microbiol.

[27] Lamoth F, Cruciani M, Mengoli C, Castagnola E, Lortholary O, Richardson M (2012). Beta-glucan antigenemia assay for the diagnosis of invasive fungal infections in patients with hematological malignancies: a systematic review and meta-analysis of cohort studies from the Third European Conference on Infections in Leukemia (ECIL-3). Clin Infect Dis.

[28] Posteraro B, Tumbarello M, De Pascale G, Liberto E, Vallecoccia MS, De Carolis E (2016). (1,3)-β-d-Glucan-based antifungal treatment in critically ill adults at high risk of candidaemia: an observational study. J Antimicrob Chemother.

[29] Nucci M, Nouèr SA, Esteves P, Guimarães T, Breda G, de Miranda BG (2016). Discontinuation of empirical antifungal therapy in ICU patients using 1,3-β-d-glucan. J Antimicrob Chemother.

[30] Richardson WS (2002). Five uneasy pieces about pre-test probabilities. J Gen Intern Med.

[31] Leeflang MMG, Bossuyt PMM, Irwig L (2009). Diagnostic test accuracy may vary with prevalence: implications for evidence-based diagnosis. J Clin Epidemiol.

[32] Bouza E, Burillo A, Muñoz P, Guinea J, Marín M, Rodríguez-Créixems M (2013). Mixed bloodstream infections involving bacteria and Candida spp. J Antimicrob Chemother.

