# Supplementary material for: Combined use of serum (1,3)-β-d-glucan and procalcitonin for the early differential diagnosis between candidaemia and bacteraemia in intensive care units
Source: Crit Care. 2017 Jul 10;21:176. doi: 10.1186/s13054-017-1763-5 (PMC5504626; doi:10.1186/s13054-017-1763-5)
Supplement: Supplementary file 1 — Fagan’s nomograms of pre-test and post-test probability of candidaemia according to BDG and PCT results. (PDF 408 kb) [file 13054_2017_1763_MOESM1_ESM.pdf]

Additional file 1. Fagan's nomograms of pre-test and post-test probability of candidaemia according to BDG and PCT results. The pre-test and post-test probability of bacteraemia can be calculated as 1 minus the pre-test or post-test probability of candidaemia, respectively.

Panel a and b. BDG results available and PCT unknown

a) BDG ≥ 80 pg/ml (PCT unknown)

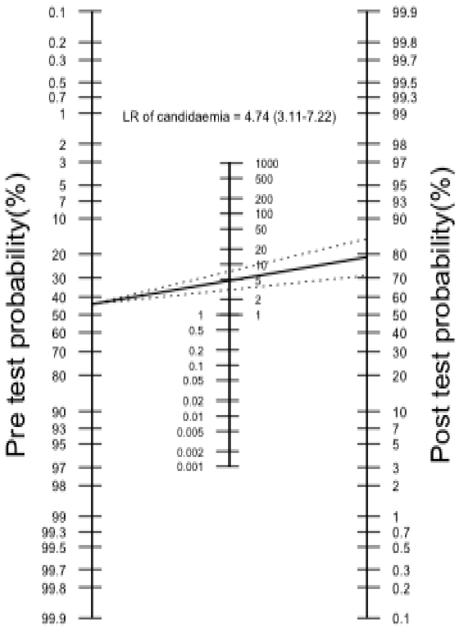

b) BDG < 80 pg/ml (PCT unknown)

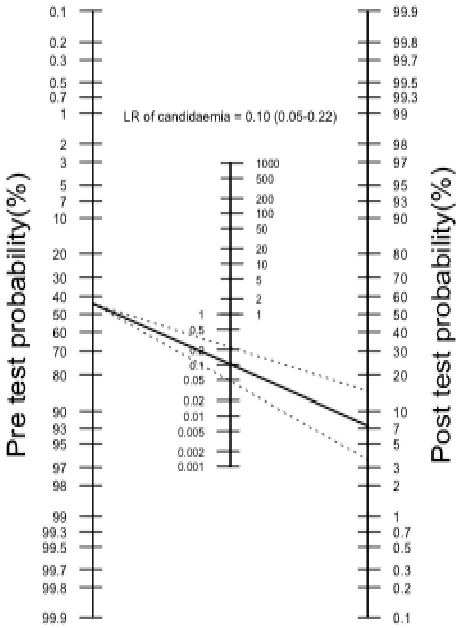

Panel c and d. PCT results available and BDG unknown

c) PCT < 2 ng/ml (BDG unknown)

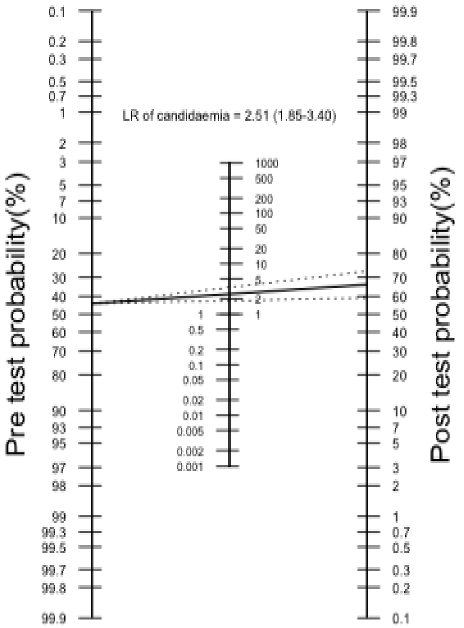

d) PCT ≥ 2 ng/ml (BDG unknown)

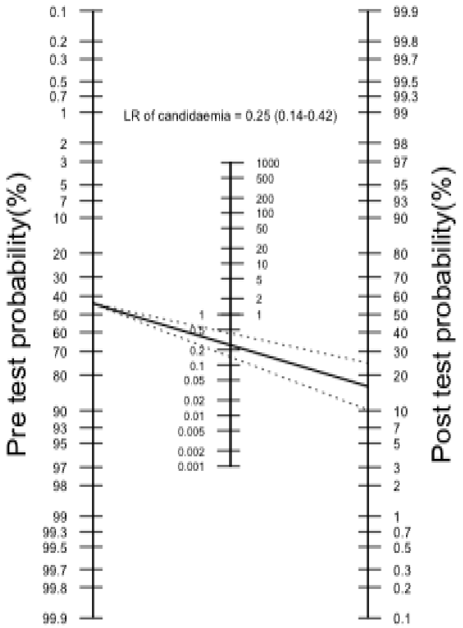

(continue)

Panel e and f. Concordant BDG and PCT results

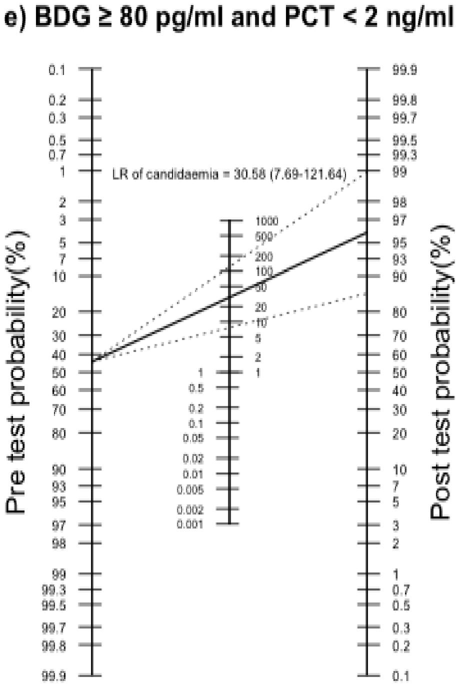

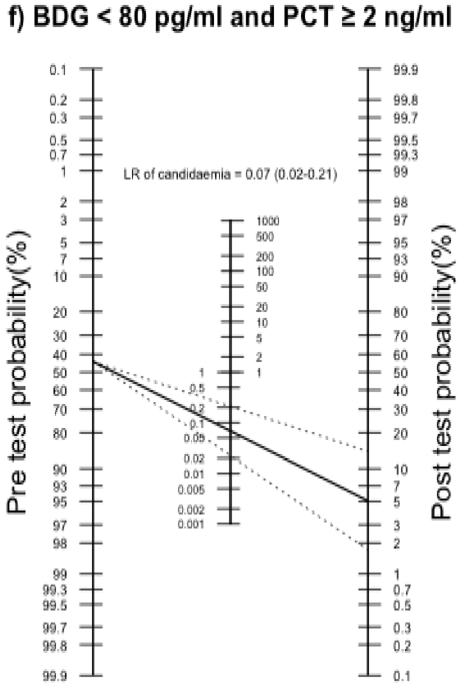

Panel g and h. Discordant BDG and PCT results

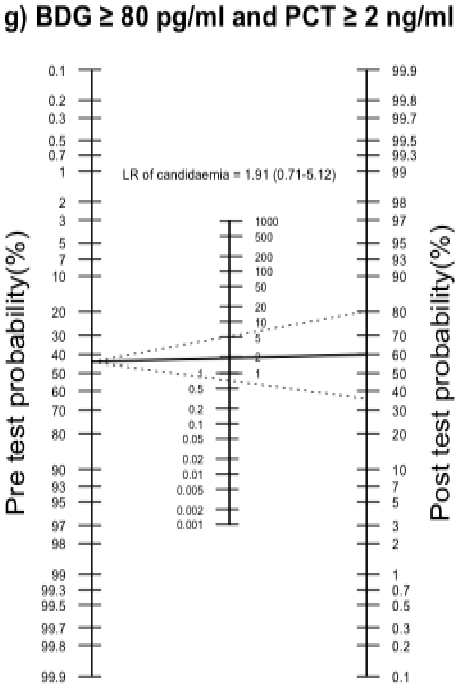

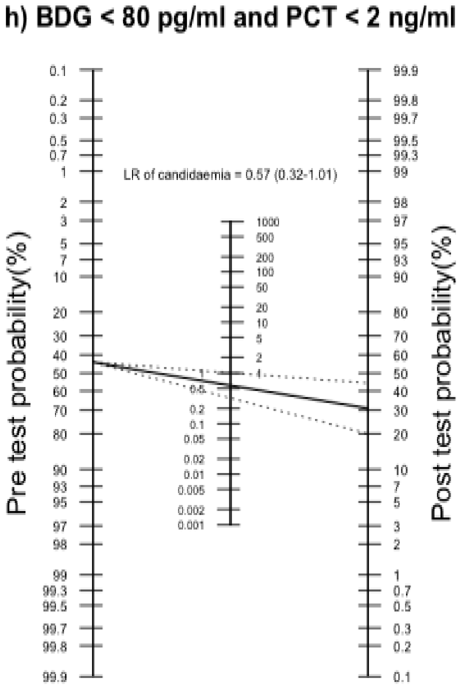

Legend. The likelihood ratio (LR) of candidemia according to the different combinations of tests results is expressed with its 95% confidence intervals (CI) in parentheses; BDG, (1,3)-ß-D-glucan; PCT, procalcitonin. Solid and dotted lines indicate changes from pre-test to post-test probability of candidaemia according to the LR of the disease and its 95% CI, respectively, at the prevalence of candidaemia registered in our population (44%). Panels a and b show the influence of BDG results on the post-test probability of candidaemia independently of PCT results. It is worth noting that both BDG ⩾ 80 pg/ml and BDG < 80 pg/ml considerably influenced

the post-test probabilities of the two diseases. A less marked but appreciable change in the post-test probability of candidaemia and bacteraemia was also observed when low (< 2 ng/ml) or high (⩾ 2 ng/ml) PCT results were considered independently of BDG (panels c and d). When the two markers were used in combination, the presence of concordant BDG and PCT results indicative of candidaemia (BDG ⩾ 80 pg/ml and PCT < 2 ng/ml) had a greater impact in favouring the diagnosis of candidaemia compared to the two markers considered separately (panel e). In contrast, when BDG and PCT concordantly indicated bacteraemia (BDG < 80 pg/ml and PCT ⩾ 2 ng/ml), their combined performance in favouring bacteraemia was very similar to that of BDG used alone (panel f). Finally, in the case of discordant BDG and PCT results, a positive or negative BDG only slightly altered the probabilities of candidaemia and bacteraemia when paired with discordant PCT values (panels g and h).
