# Supplementary material for: Combined use of serum (1,3)-β-d-glucan and procalcitonin for the early differential diagnosis between candidaemia and bacteraemia in intensive care units
Source: Crit Care. 2017 Jul 10;21:176. doi: 10.1186/s13054-017-1763-5 (PMC5504626; doi:10.1186/s13054-017-1763-5)
Supplement: Supplementary file 2 — Example of a possible therapeutic model based on Fagan’s nomograms of pre-test and post-test probability of candidaemia and bacteraemia according to BDG and PCT results. (PDF 382 kb) [file 13054_2017_1763_MOESM2_ESM.pdf]

Additional file 2. Example of a possible therapeutic model based on Fagan's nomograms of pre-test and post-test probability of candidaemia and bacteraemia according to BDG and PCT results*

***Suggestions are based on our provisory retrospective results; the model is intended to be used for research purposes only and not for guiding therapy, pending confirmatory studies.**

Legend: LR, likelihood ratio; 95% CI, 95% confidence intervals; BDG, (1,3)-ß-D-glucan; PCT, procalcitonin; PPC, post-test probability of candidaemia; PPB, post-test probability of bacteraemia.

Possible therapeutic suggestions on the basis of tests results were expressed according to the different PPC and PPB obtained on Fagan's nomograms by intersecting the limits of the 95% CI of the LR of candidaemia at any possible prevalence of candidaemia rounded to the nearest 5%, and by defining probability of bacteraemia as 1 – probability of candidaemia. Detailed criteria for therapeutic suggestions were as follows: PPC < 5%, do not initiate early antifungals/discontinue empiric antifungals (if stable conditions and clinical picture not indicative of candidaemia); PPC 5-20%, manage antifungals according to clinical judgment; PPC >20%, initiate early antifungals/prosecute empiric antifungals; PPB < 5%, do not initiate early antibacterials/discontinue empiric antibacterials (if stable conditions and clinical picture not indicative of bacteraemia); PPB 5-20%, manage antibacterials according to clinical judgment; PPB >20%, initiate early antibacterials/prosecute empiric antibacterials. Rounded prevalences equal to 0% and 100% were not considered for defining thresholds.

A) Results of tests indicative of candidaemia

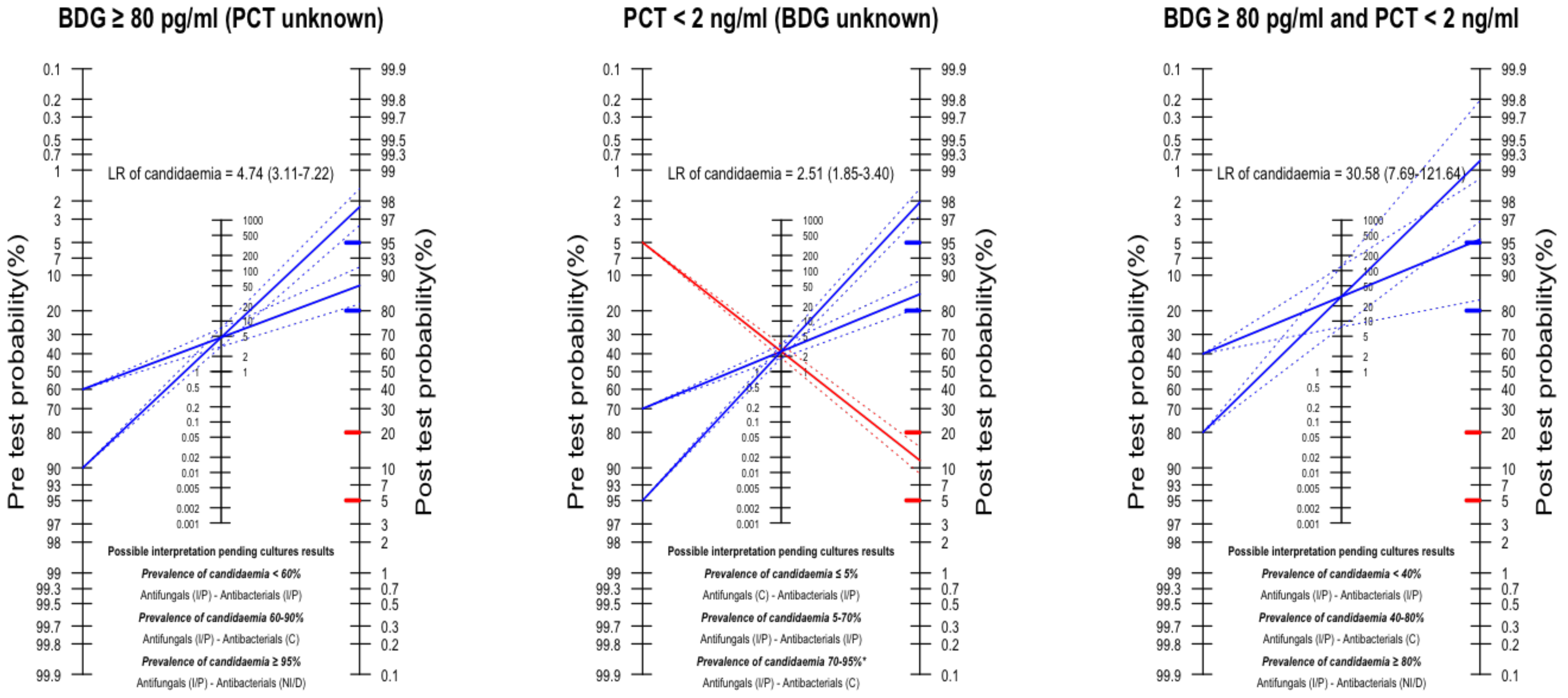

Legend. Solid and dotted blue lines indicate changes from pre-test to post-test probability of candidaemia according to the LR of the disease and its 95% CI, respectively, at the prevalence thresholds (pre-test probabilities) that differentiated suggestions about the use of antibacterials. Solid and dotted red lines indicate changes from pre-test to post-test probability of candidaemia according to the LR of the disease and its 95% CI, respectively, at the prevalence thresholds (pre-test probabilities) that differentiated suggestions about the use of antifungals. I/P, initiate/prosecute; NI/D, not initiate/discontinue; C, manage according to clinical judgment. *In the case of PCT < 2 ng/ml, BDG unknown, and prevalence of candidaemia ≥ 95%, possible interpretation pending cultures results is as follows: Antifungals (I/P) - Antibacterials (NI/D).

B) Results of test indicative of bacteraemia

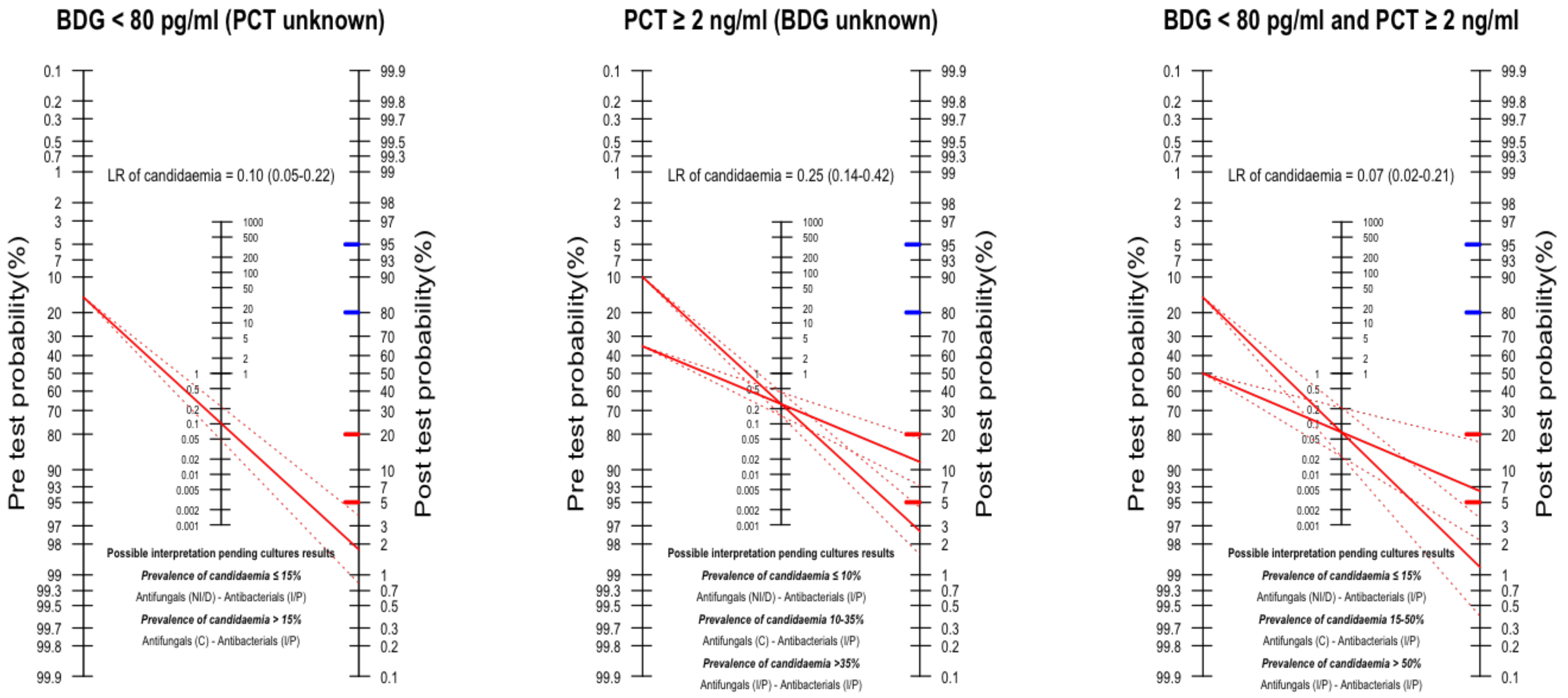

Legend. Solid and dotted blue lines indicate changes from pre-test to post-test probability of candidaemia according to the LR of the disease and its 95% CI, respectively, at the prevalence thresholds (pre-test probabilities) that differentiated suggestions about the use of antibacterials. Solid and dotted red lines indicate changes from pre-test to post-test probability of candidaemia according to the LR of the disease and its 95% CI, respectively, at the prevalence thresholds (pre-test probabilities) that differentiated suggestions about the use of antifungals. I/P, initiate/prosecute; NI/D, not initiate/discontinue; C, manage according to clinical judgment.

C) Discordant results of BDG and PCT

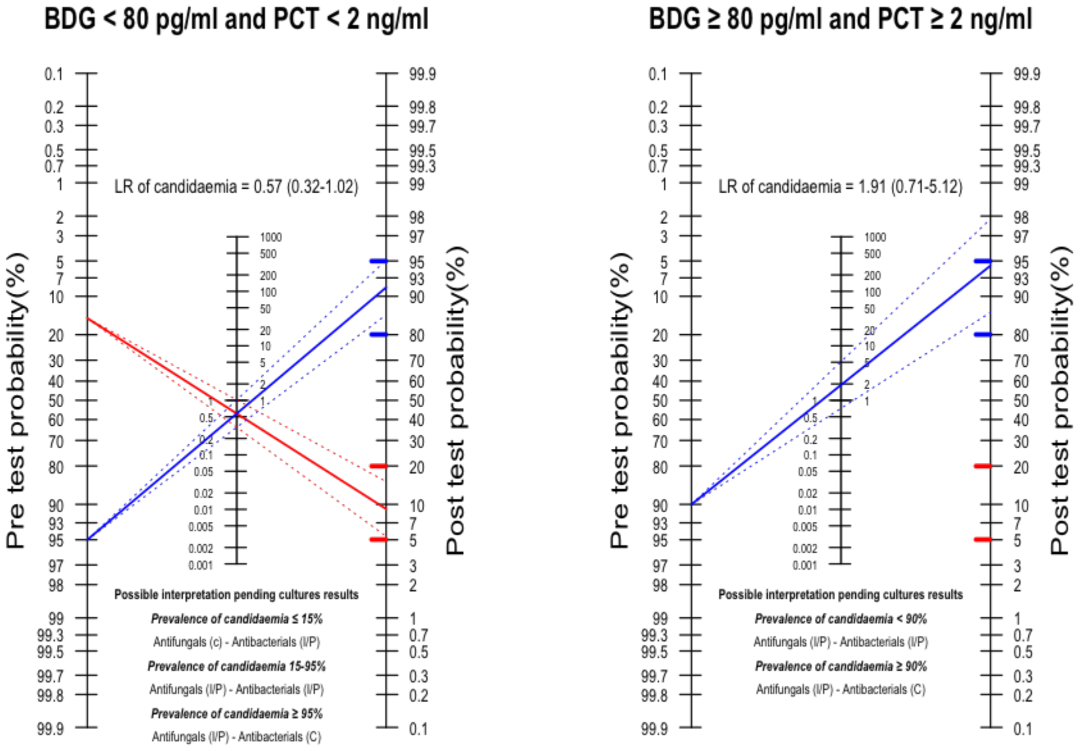

Legend. Solid and dotted blue lines indicate changes from pre-test to post-test probability of candidaemia according to the LR of the disease and its 95% CI, respectively, at the prevalence thresholds (pre-test probabilities) that differentiated suggestions about the use of antibacterials. Solid and dotted red lines indicate changes from pre-test to post-test probability of candidaemia according to the LR of the disease and its 95% CI, respectively, at the prevalence thresholds (pre-test probabilities) that differentiated suggestions about the use of antifungals. I/P, initiate/prosecute; NI/D, not initiate/discontinue; C, manage according to clinical judgment.
